# Supplementary material for: CLDN6 Suppresses c–MYC–Mediated Aerobic Glycolysis to Inhibit Proliferation by TAZ in Breast Cancer
Source: Int J Mol Sci. 2021 Dec 23;23(1):129. doi: 10.3390/ijms23010129 (PMC8745066; doi:10.3390/ijms23010129)
Supplement: Supplementary file 1 [file ijms-23-00129-s001.zip › ijms-1486225-supplementary.pdf]

Supplementary Figure S1

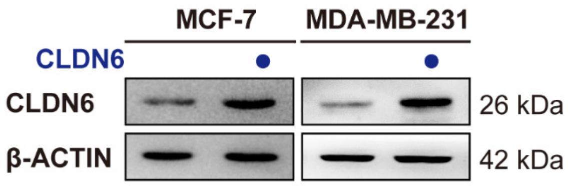

Figure S1. WB validation of CLDN6 overexpression in MCF-7 and MDA-MB-231 cells. **Supplementary Figure S2**

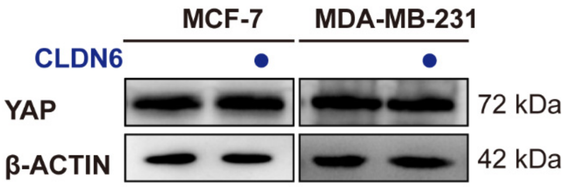

Figure S2. WB analysis of the effect of CLDN6 on YAP expression in MCF-7 and MDA-MB-231 cells.

Supplementary Table S1 The first antibodies used in IHC

Name	Catalog Number	Manufacturer	Dillution
CLDN6	sc-393671	Santa Cruz, USA	1:100
TAZ	OTI1H9	Origene, USA	1:150
c-MYC	RMA-0803	MXB Biotechnology, CN	Ready to use
GLUT1	21829-1-AP	Proteintech, CN	1:1000
LDHA	19987-1-AP	Proteintech, CN	1:100

Supplementary Table S2 The primers used in RT-PCR

Genes	Primer Sequence
β-ACTIN	5′-TCATGAAGTGTGACGTGGACATC-3′<br>5′-CAGGAGGAGCAATGATCTTGATCT-3′
CLDN6	5′-TTCATCGGCAACAGCATCGT-3′<br>5′-GGTTATAGAAGTCCCGGATGA-3′
c-MYC	5′-AGAGTCTGGATCACCTTCTGCTGGA-3′<br>5′-ACATTCTCCTCGGTGTCCGAGGAC-3′
GLUT1	5′-CAGGAGATGAAGGAAGAGAGTCG-3′<br>5′-CGAAGATGCTCGTGGAGTAATAG-3′
LDHA	5′-CCAGTTTCCACCATGATTAAGGG-3′<br>5′-GAAGTCAGAGTCACCTTCACAAG-3′

Supplementary Table S3 The first antibodies used in WB

Name	Catalog Number	Manufacturer	Dilution
β-ACTIN	66009-1-Ig	Proteintech, CN	1:2000
CLDN6	sc-393671	Santa Cruz, USA	1:500
c-MYC	10828-1-AP	Proteintech, CN	1:1000
GLUT1	21829-1-AP	Proteintech, CN	1:1000
LDHA	19987-1-AP	Proteintech, CN	1:2000
TAZ	A15806	Abclonal, CN	1:1000
YAP	A19134	Abclonal, CN	1:1000

Supplementary Table S4 The first antibodies used in IF

Name	Catalog Number	Manufacturer	Dillution
CLDN6	sc-393671	Santa Cruz, USA	1:100
TAZ	A15806	Abclonal, CN	1:100
